# Optimization of incubation temperature in embryonated chicken eggs inoculated with H9N2 vaccinal subtype of avian influenza virus

**Published:** 2013

**Authors:** Iraj Khalili, Rahim Ghadimipour, Ali Ameghi, Saeed Sedigh-Eteghad

**Affiliations:** *Department of Research and Development, Razi Vaccine and Serum Research Institute, Marand, Iran.*

**Keywords:** Inactivated influenza vaccine, Incubation temperature, Optimization

## Abstract

There are little information about growth properties of low pathogenic (LP) avian influenza virus (AIV) in embryonated chicken eggs (ECEs) at different incubation temperatures. Knowledge of this information increases the quantity and quality of antigen in vaccine production process. For this purpose, 10^-5^ dilution of AIV (A/Chicken/Iran/99/H9N2) was inoculated (Intra-allantoic) into 400, 11-day old specific pathogen free (SPF) ECEs in the 0.1 mL per ECE rate and incubated in 32, 33, 34, 35, 36, 37.5, 38, 39 ˚C for 72 hr in 65% humidity. Early death embryos in first 24 hr were removed. Amnio-allantoic fluid was withdrawn into the measuring cylinder, and tested for hemagglutination (HA) activity and egg infective dose 50 (EID_50_). The utilizable ECEs and amnio-allantoic fluid volume was significantly increased in 35 ˚C, (*p *< 0.05). Significant difference in HA and EID_50_ titers, were seen only in 39 ˚C group. Therefore, 35°C is an optimum temperature for incubation of inoculated ECEs.

## Introduction

Among the avian influenza virus (AIV) subtypes, H9N2 virus has the potential to cause an influenza pandemic because of its wide prevalence in avian species and the ability to infect humans.^   1 ^ An outbreak of H9N2 infection in poultry farms was first reported in 1998 in a layer farm in Tehran-Iran.^2^^,^^3^ At the same time the similar respiratory disease outbreaks were observed in broiler and breeder flocks located in Tehran and neighbor provinces.^   2 ^

This disease is now endemic and vaccination against this subtype is practiced, routinely.^   4 ^ This virus cause mild disease among terrestrial birds. However, it can also cause severe outbreaks in poultry depending on the circumstances.^        5 ^

Nowadays, two types of influenza vaccines are approved for clinical use, and both of them are manufactured in embryonated chicken eggs (ECEs): live attenuated influenza vaccines and inactivated influenza vaccines.^6^^, ^7

Inactivated influenza vaccine subtype H9N2 production was started in Iran in 1998 and nowadays this product is one of best inactived vaccines from quality and quantity point view.^   8 ^

The ECE methods are widely used in inactivated H9N2 whole-virus vaccines manufacturing^   9 ^ and this kinds of vaccines were shown to bear better immunogenic responses than other vaccine types.^   6^

For the production of vaccines the growth characteristics of the vaccine strains and the yields of the viral antigens (quantity and quality) are important issues.^   10 ^ The incubation temperature of inoculated ECEs is closely associated with quantity and quality of antigen in vaccine production process. Therefore, the number of utilizable ECEs and total volume of yielded antigen are very important factor from economical point view. 

The effects of different incubation temperatures on some subtypes of AIV replication has been well studied in various infection systems, including cultured cells and animal models. But there are little information about the growth properties of low pathogenic (LP) H9N2 AIVs in ECEs at different incubation temperatures.^   11 ^

Therefore, optimization of ECEs incubation temperatures, in H9N2 vaccine production process seems necessary for reduction of extra production costs and increase amount of vaccine output. 

## Material and Methods

**Virus preparations.** Standard vaccine strain AIV (A/Chicken/Iran/99/H9N2) was used to inoculate 11-day old specific pathogen free ECEs. The eggs were observed for 24-72 hr post inoculation. Allantoic fluid of the inoculated eggs were collected and centrifuged at 1200 rpm for 30 min and supernatant were assayed. 

Egg infective dose 50 (EID_50_) of the samples was calculated by the method of Reed and Muench in SPF eggs with 11-day-old embryos.^   12 ^

Hemagglutination assay (HA) was performed in V-bottom 96-well plates with 1% chicken red blood cell, as described with Burleson *et al*.^   13^

**Study design. **Prepared 10^-5^ diluted working seed of AIV (A/Chicken/Iran/99/H9N2) solution (EID_50 _= 9.8 log_10_ and HA titer = 10 log_2_) was inoculated into 400 SPF ECEs at the rate of 0.1 mL per ECE, via intra-allantoic way. All eggs were sealed with wax. Inoculated eggs were randomly divided into 8 groups (n = 50) and incubated in various temperatures. The experimental temperatures were 32, 33, 34, 35, 36, 37.5 (standard temperature), 38 and 39 ˚C. Inoculated ECEs were incubated for 72 hr with 65% humidity. Eggs were candled daily and early death embryos in first 24 hr were removed. 

In the next step, the eggs were chilled at 4 ˚C for 24 hr to kill the embryo and to reduce the contamination of the allantoic fluid with blood during harvesting.

In harvesting stage, after removing the shell and shell membranes at the blunt end of the eggs, the amnio-allantoic fluid samples were withdrawn into the small measuring cylinders by pressing the suction bulb until the total volume of each egg allantoic liquid was obtained. Allantoic fluid from surviving embryos was tested for HA activity and EID_50_. Assays were performed in three replicates.

**Statistical analysis.** Data were analyzed using SPSS (version 17 for Windows, SPSS Inc., Chicago, IL, USA), and comparisons were made using the descriptive statistics and one way ANOVA tests.

## Results

The utilizable ECEs and amnio-allantoic fluid volume was noticeably influenced by some temperatures. Amount of utilizable ECEs in 33 and 35 ˚C incubation temperature groups were significantly higher than standard incubation temperature (37.5 ˚C) and the greatest total antigen volume of 609.00 ± 70.37 mL occurred under 35 ˚C incubation temperature, that was greater than other experimental groups (*p* < 0.05).

Mean of HA titers and EID_50_ tests in the study groups are shown in Table 1. No significant differences were seen for mentioned tests between groups. Only in 39 ˚C incubation temperature, HA titer and EID_50_ test results were significantly decreased.

**Table 1:** Interaction of incubation temperature with antigen volume and quality factors (Three replicates mean values ± SD).

**Incubation temperature (˚C) **	**Early death embryos (no.)**	**Utilizable ** **eggs (no.)**	**Total antigen volume (mL)**	**Antigen volume (mL)** ** / Utilizable eggs**	**Antigen volume ** **(mL) / All eggs**	**HA** **Log**_2_	**EID50** **Log**_10_
** 32 **	7.33 ± 2.51	42.66 ± 2.51	470.00 ± 67.26	10.98 ± 0.97	9.40 ± 1.34	9.02 ± 0.21	9.46 ± 0.06
** 33 **	6.33 ± 2.08*	43.66 ± 2.08*	536.66 ± 31.75*	12.29 ± 0.82	10.73 ± 0.63*	8.77 ± 0.19	9.66 ± 0.16
** 34 **	7.33 ± 2.08	42.66 ± 2.08	560.00 ± 28.61*	13.12 ± 0.13	11.18 ± 0.58*	9.22 ± 0.25	9.54 ± 0.52
** 35 **	4.66 ± 2.08*	45.33 ± 2.08*	609.00 ± 70.37*	13.45 ± 1.69	12.18 ± 1.40*	9.44 ± 0.95	9.55 ± 0.09
** 36 **	11.33 ± 3.21	38.66 ± 3.21	480.00 ± 61.44	12.45 ± 1.64	9.60 ± 1.22	9.30 ± 0.67	9.49 ± 0.18
** 37.5 **	13.33 ± 1.15	36.66 ± 1.15	385.66 ± 65.54	10.50 ± 1.66	7.71 ± 1.31	9.13 ± 0.55	9.06 ± 0.35
** 38 **	17.00 ± 2.00	33.00 ± 2.00	299.66 ± 57.70	9.02 ± 1.19	5.99 ± 1.15	8.91 ± 1.04	8.25 ± 0.64
** 39 **	22.00 ± 2.64*	28.00 ± 2.64*	248.66 ± 5.13	8.91 ± 0.65	4.97 ± 0.10	5.50 ± 0.25*	7.13 ± 0.27*

* indicates significant differences compared to 37.5 ˚C (Standard temperature) at *p *< 0.05.

## Discussion

All avian eggs necessarily lose water because of porous eggshell.^   14 ^ About 10-11% of the water in an embryo is lost during the incubation period.^   15 ^ Some studies show that, the change in volume and composition of amnio-allantoic fluids are closely associated with water metabolism of avian egg.^   16 ^ Reportedly, the phase change from liquid water to water vapor requires heat and every extra calorie/hr increase the amount of evaporation and water lost.^   17 ^

Romanoff and Hayward showed that extreme temperatures (39.5 and 34.5 ˚C) significantly reduced the amnio-allantoic fluids^   16 ^ but in the present study, the best result in antigen volume (mL) per utilizable ECEs in vaccine manufacturing process was achieved in 35 ˚C incubation temperature.

On the other hand, the mortality of inoculated ECEs 24 hr post-inoculation is generally considered non-specific^   18 ^ and according to the Office International des Epizooties (OIE) manual should be discarded.^   19 ^ Therefore, increasing of first 24 hr mortality can affect the quality and economic factors in vaccine industry. 

Our results showed that temperature variation was the effective factor in this rate; utilizable eggs in the 35 ˚C incubation temperature were significantly higher than standard incubation temperature. 

The rapid replication of vaccine virus with highly active HA and neuraminidase may be a burden to embryos, and may cause early embryonic mortality. High embryonic virulence may result in a decrease of the amount of allantoic fluid and an increase in the number of discarded ECEs.^   20 ^ Lang *et al.* showed that avian influenza virus can be isolated from ECE within 48 hr by incubation at 37 ˚C or 39 ˚C, whereas 72 hr is required if ECE are incubated at 35 ˚C.^   11 ^ Also, Hahon *et al.* study results showed that an incubation temperature of 35 ˚C is favored for the multi-plication of virus on the chorio-allantoic membrane of ECEs, while higher temperatures are found to be less optimal.^   21 ^

In the present paper the HA titer and EID_50_ test were evaluated in different incubation temperatures for prepared AIV and there were not any significant differences between treatment groups (except 39 ˚C group). Also in Lang *et al.* study there are no statistically significant differences in the mean HA titers and EID_50_ of low pathogen avian influenza viruses (LPAIV) at different incubation temperatures.^   11 ^ However, in the Blumenthal *et al.* work, the HA titers are the highest and best sustained in ECEs incubated at 34 ˚C,^   22 ^ that may be related with the virus strain. 

Therefore, 35 ˚C is an optimum temperature for incubation of inoculated ECEs, in inactivated AI (A/Chicken/ Iran/99/H9N2) vaccine production process and this optimization help reduce extra production costs and increase amount of vaccine output. 
